# Impact of energy availability and physical activity on variation in fertility across human populations

**DOI:** 10.1186/s40101-023-00318-3

**Published:** 2023-02-24

**Authors:** Srishti Sadhir, Herman Pontzer

**Affiliations:** 1 Department of Evolutionary Anthropology, Duke University, Durham, NC USA; 2 Duke Global Health Institute, Duke University, Durham, NC USA

**Keywords:** Maternal energetics, Reproduction, Fertility, Resource availability, Physical activity

## Abstract

Human reproduction is energetically costly, even more so than other primates. In this review, we consider how the energy cost of physical activity impacts reproductive tasks. Daily energy expenditure appears to be constrained, leading to trade-offs between activity and reproduction expenditures in physically active populations. High workloads can lead to suppression of basal metabolic rate and low gestational weight gain during pregnancy and longer interbirth intervals. These responses lead to variation in fertility, including age at first reproduction and interbirth interval. The influence of energetics is evident even in industrialized populations, where cultural and economic factors predominate. With the decoupling of skills acquisition from food procurement, extrasomatic resources and investment in individual offspring becomes very costly. The result is greater investment in fewer offspring. We present a summary of age at first reproduction and interbirth interval trends across a diverse, global sample representing 44 countries and two natural fertility populations. While economic factors impact fertility, women in energy-rich, industrialized populations are capable of greater reproductive output than women in energy-stressed populations. Thus, energetic factors can be disentangled from cultural and economic impacts on fertility. Future research should focus on objective measurements of energy intake, energy expenditure, and physical activity in a broader sample of populations to elucidate the role of energetics in shaping reproductive outcomes and health.

**Supplementary Information:**

The online version contains supplementary material available at 10.1186/s40101-023-00318-3.

## Introduction

Reproduction is energetically costly for primates and uniquely so for humans. Humans have evolved longer gestation lengths and larger neonates than other hominoids and consequently expend more energy in reproduction than our closest living evolutionary relatives [1, 2]. The evolution of hunting and gathering in the hominin lineage has also increased the daily energy costs of physical activity, as adults in hunter-gatherer communities walk farther and work harder to acquire food than apes in the wild [3–8]. The dual energetic challenges of increased reproductive and activity demands have shaped the evolution of human physiology and continue to influence variation in fertility across human populations [9–15]. In this review, we examine the impact of physical activity workload on fertility variation across human populations. While variation in fertility today is heavily shaped by cultural and economic factors affecting women’s access to education and contraception, the effects of energetics are still evident. We examine current evidence for the influence of energetics on fertility patterns between and within human populations and discuss directions for future research.

## Energy balance and evolutionary constraint

Compared to other hominoids, human reproduction is remarkably cooperative. As Hawkes and others have recognized, the energetic demands on mothers to support pregnancy, lactation, and childcare are subsidized in human populations by the contribution of food, care, and other help from other adults [5, 16, 17]. These behavioral adaptations improve food availability and can reduce physical activity workload for mothers and account for humans’ short interbirth interval (IBI) compared to other hominoids [16, 17]. Still, mothers in hunter-gatherer and farming communities are often physically active throughout pregnancy and lactation [1, 18–20], and the energy costs of reproduction are substantial.

In principle, mothers could meet the heightened energy demands of human reproduction and physical activity by simply eating more food and operating with a larger energy budget of calorie consumption and expenditure. Studies of daily energy expenditure, measured using the doubly labeled water method, lend some support to this scenario. Humans have evolved larger energy budgets than chimpanzees, bonobos, gorillas, and orangutans, consuming and expending more energy per day than other apes in analyses controlling for body size [2]. During pregnancy and lactation, daily expenditures for mothers increase even further, particularly in populations that are well-nourished and relatively sedentary [21–23].

Other studies point to potential constraints on energy budgets, suggesting the capacity to increase daily expenditures is limited. Across human populations, adults (both men and women) have broadly similar size-adjusted daily energy expenditures despite wide variation in daily physical activity, suggesting daily energy expenditures operate under some evolved constraint [24–29]. Similar constraint is evident in other mammals as well [30–33]. Analyses of extreme endurance events (e.g., ultramarathons, Tour de France, arctic trekking) suggest that pregnancy pushes human physiology to the limits of daily energy expenditure [34]. These analyses further suggest that human physiology is constrained by the rate that food energy can be assimilated, estimated to be approximately 2.5 times a person’s basal metabolic rate (BMR, the rate of expenditure at rest, fasted, and in a thermoneutral environment) [34]. Ellison, Dunsworth, and others have argued that human metabolic constraints limit the length of gestation and maximal physiological investment into reproduction [9, 10, 35, 36].

Energy budgets can be further constrained through extrasomatic, nonphysiological factors, such as food insecurity. Researchers overseeing a maternal nutritional intervention program in The Gambia in the 1980s identified energy-sparing metabolic adaptations (i.e., BMR suppression) that reduced the total energy cost of pregnancy in undernourished mothers [37–41]. Maternal metabolism can respond to low food availability by suppressing BMR early in pregnancy and reducing gestational weight gain (particularly maternal fat mass), keeping energy expenditure in check to match energy intake (3–7). In doing so, maternal physiology protects the fetus against macronutrient fluctuations and deficiencies in utero (i.e., Maternal Nutritional Buffering Model) [42]. Mechanisms of energy sparing might also be predicted in highly active women in industrialized populations who are food secure but are operating at the presumed limit to energy absorption and assimilation due to their high physical activity workload.

Constraints on the daily energy budget suggest humans cannot always increase intake and expenditure to meet the demands of physical activity and reproduction. Instead, for mothers approaching the physiological limits on daily expenditure, increased physical activity may lead to trade-offs in energy allocation, reducing energy expended in other tasks, including reproduction. These trade-offs, in turn, may influence variation in fertility.

## Physical activity, energy status, and fertility

Much of the work examining variation in fertility across populations from an ecological perspective has focused on female “energy status,” the balance of energy expended in physical activity, and other tasks against food energy availability and fat stores [12, 27]. Many of these studies, particularly foundational work from the 1980s and 1990s, lacked direct, objective measures of daily energy expenditure or physical activity. Nonetheless, the results from this work are broadly consistent with the perspective that energy budgets are limited, and that physical activity trades off against investment in reproduction. In women with low energy status due to high physical activity workload, reproduction is often delayed, slowed, or temporarily compromised [10]. Conversely, when women’s energy status is improved through the introduction of work-saving technologies, fertility often increases [43–45].

The underlying mechanisms for these energetic effects have been well-described in work examining the hypothalamic–pituitary–adrenal axis (HPAA) [10, 27, 46]. Nutritional status was associated with suppressed ovarian function and low progesterone levels in Lese women, consistent with low fertility rates in this subsistence farming population [47, 48]. Similarly, positive energy balance (i.e., weight gain) during postpartum lactation promotes ovarian function and an earlier resumption of menstrual cycling in forager-horticulturalist Qom women independent of nursing intensity [49, 50].

Seasonal changes in physical activity workload have also been shown to influence reproductive physiology within populations. Seasonality in births is evident among Gambian women, where the lowest number of births occur at the beginning of the wet season just after the agricultural season in which women have high workloads and the lowest energy intake [51, 52]. This pattern is also evident among Nepali women, who experience suppressed ovarian function and reduced fecundability during the monsoon season, when subsistence workload is the most demanding [15]. In a sample of agricultural Polish women who do not experience nutritional stress, progesterone levels were suppressed in the summer months with the highest agricultural workload [53]. Low nutritional status and high workload are both indicative of a low metabolic resource environment, in which women may not have enough food and/or must continue to actively contribute to market and household labor through gestation and postpartum lactation.

Changes to female reproductive life history have been noted with cultural changes in sedentarization and reduced physical activity [54, 55]. In a comparison of the energetics of food acquisition, Kraft and colleagues showed that subsistence farming generally reduces women’s daily physical labor, consistent with the trend toward higher total fertility rates among farmers [3]. Indeed, the adoption of agriculture during the Neolithic transition is associated with a ~15% to 20% increase in estimated fertility, based on analyses of archeological skeletal collections [54]. Today, women in natural fertility, small-scale populations undergoing market integration (subsistence farming to mixed market economy) or adopting labor-saving subsistence technologies, initially see enhancement in total fertility rate (TFR) [43–45, 55]. In Agta hunter-gatherers of the Philippines, high fertility is achieved during initial sedentarization at the expense of offspring survival [55]. High-fertility populations undergoing demographic transition therefore see a trade-off between offspring quality and quantity [55, 56].

## Energetics and reproduction in industrialized societies

If energy was the only factor influencing fertility, industrialization would be associated with increased family size. The mechanization of food production and other work activities has greatly reduced daily physical activity in economically developed countries while simultaneously increasing the availability of calorie-rich foods [57]. These changes presumably improve the energy status of women but are not associated with an increase in TFR. Instead, as is well known, industrialization and economic development are associated with a demographic transition to smaller desired family sizes and later age at first reproduction (AFR). Today, industrialized populations generally have much lower TFR than natural fertility, small-scale populations [58–60].

Fertility in post-demographic transition, industrialized populations reflects non-energetic proximate factors mediated by contraceptive use: smaller ideal family size and changing social norms, education level, income, government policies, and other socioeconomic factors [61–63]. Human capital theory [64], and by extension, embodied capital theory [65], invoke an ultimate explanation for low fertility rates in industrialized population. Human capital is described as activities which increase resources in individuals for economic gains [64]. These activities include accruing knowledge, habits, and values that will contribute to future economic achievement [64]. Embodied capital theory integrates human capital theory, which was developed in economics, with life history theory in biology. Embodied capital theory proposes that human foraging activities required the development of complex skills that take a long childhood and adolescent period to master. As a result, human physiology and psychology have evolved to favor extended juvenile periods and greater investment in each offspring [56, 65]. For pre-industrial populations, including subsistence farmers and hunter-gatherers, skills are linked to food procurement, thereby providing a direct link to energetics and fertility outcomes. In industrialized populations with abundant extrasomatic wealth beyond simply food resources, skills acquisition moves beyond food procurement, motivating a delay in fertility.

For women in industrialized populations, extrasomatic resource acquisition (education attainment and income) and investment in offspring are very costly [66]. Therefore, while women in industrialized populations have the energetic means (via high nutritional status, low immune burden, low physical activity levels, etc.) to invest in more offspring, the cost of attaining extrasomatic resources is high, and raising high-quality offspring means reducing offspring numbers and investing more heavily in each individual offspring. These costs are likely shaped by perceived education and income attainment capabilities and government policies and influence social norms about reproduction and ideal family size. It is worth noting that women from the United States (US) and other industrialized populations routinely fall short of their intended number of children due to high investment costs [62, 66].

Despite the predominance of cultural and economic influences on reproductive behavior, the influence of energy status may still be evident. For example, economic development is associated with a weaker contraceptive effect of breastfeeding and a shortening of the IBI among populations worldwide [67]. The energy stress of breastfeeding is an essential component of its suppressive effects on ovarian function [10, 12]. The reduced contraceptive effect of breastfeeding with economic development is consistent with a reduction in physical activity workload and a subsequent increase in the energy available for reproduction. Shorter periods of lactational amenorrhea indicate that women in industrialized populations have the physiological capacity for shorter IBI and greater TFR than women in subsistence populations.

## Fertility and energetics within populations

Energetic effects on fertility may also be evident between demographic groups within developed populations. Cultural and economic influences on fertility are experienced unevenly. Some subpopulations with less access to education, with lower income, or facing poverty may respond with different reproductive behaviors.

Variation in fertility within the US provides an informative example. Despite classification of the US as a high-income country [68], by some measures of poverty [69], Americans who are lower on the socioeconomic ladder fall alongside countries classified as low- or middle-income in quality of life [68, 70]. Pregnant women whose household income falls below 185% of the Federal Poverty Guidelines are eligible for the Special Supplemental Nutrition Program for Women, Infants, and Children (WIC) to ensure nutritional well-being during gestation, postpartum, and early childhood [71, 72]. As of 2016, 39.6% of US women used the program [71].

There has been considerable economic and policy interest on the impacts of WIC, but most literature on the subject is concerned with immediate health-related outcomes, such as the impact of WIC on gestational weight gain, gestational length, prenatal care initiation, birthweight, and breastfeeding practices [73–75], or child outcomes, such as growth, development, and childhood experiences [76, 77]. One study by Hoynes and colleagues [75] found no impact of WIC on fertility rate, measured as total births per 1000 women aged 15–44. However, this study did not examine differences in the proximate determinants of fertility, such AFR or IBI.

**Fig. 1:**
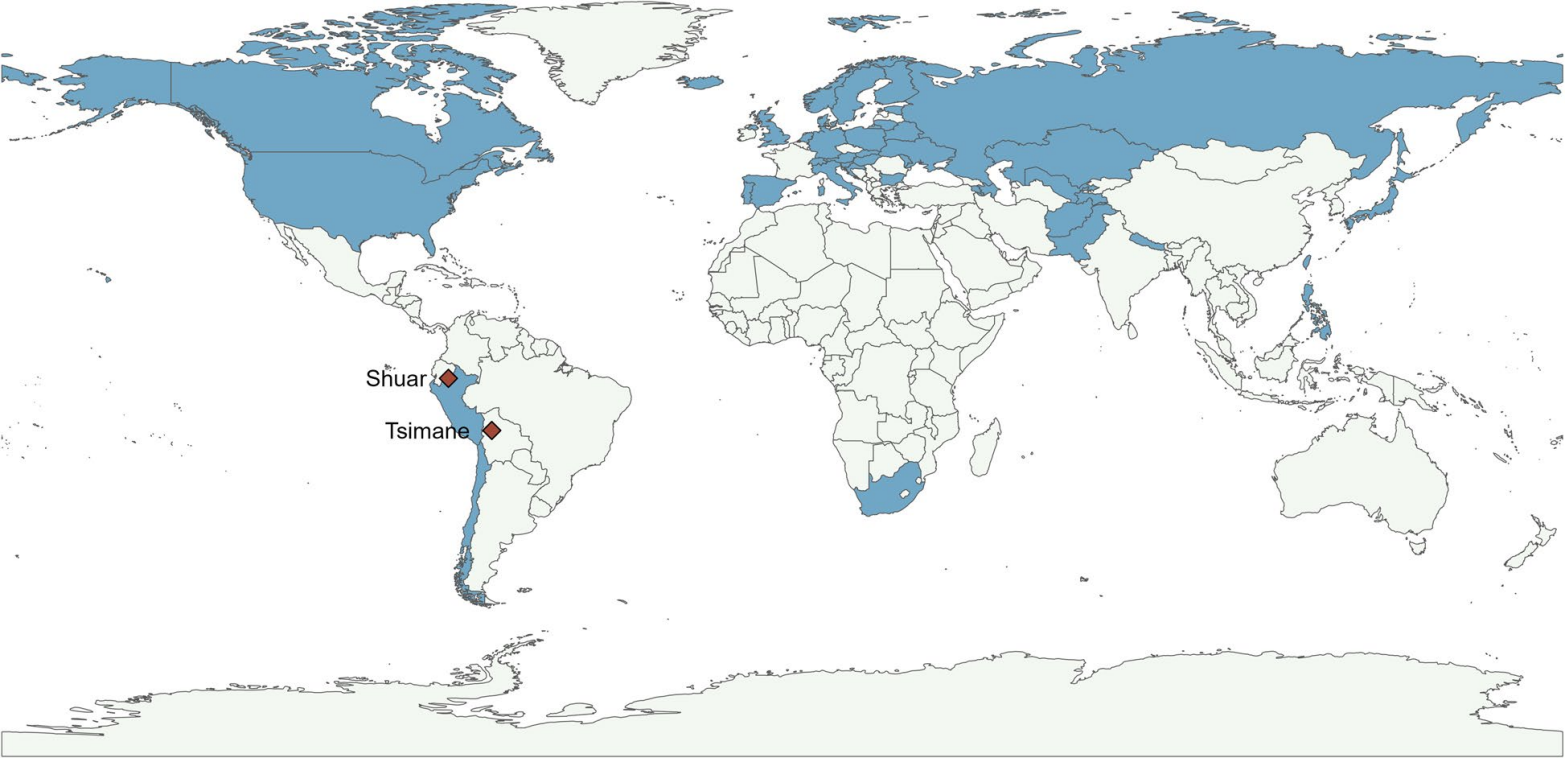
Global distribution of *n* = 44 countries and *n* = 2 natural fertility populations. Countries shaded in blue are those included in the sample. Natural fertility populations (Shuar and Tsimane) are labeled with red diamonds

We examined published survey datasets reporting individual women’s fertility and birth outcomes [78–82] across 44 countries and two small-scale, natural fertility populations [78, 80] (Fig. 1, Table S1): the Tsimane of Bolivia, who practice a mixed subsistence tradition of horticulture and hunting and gathering, and the Shuar of Ecuador, who practice hunting and gathering [58, 78] (Fig. 1, Table S2). The US data was divided by WIC status [71]. We compiled all data into two datasets: AFR (*N* = 4,984,026 women) and IBI (*N* = 840,063 women), with IBI data constrained to women ages 20–29. A total of 6 income groups were available for analysis: natural fertility (subsistence economy: Shuar and Tsimane), low, middle, and high (country economic development classification based on Gross National Income, GNI, per capita in US dollars) [68], and, for US mothers, WIC participant or non-participant. IBI values represent closed birth intervals only, with number of months calculated by date of most recent birth minus date of the next previous birth. IBI data was not available for high-income populations except for the US, so this income group was not included in the IBI analysis.

To investigate the impact of income on AFR, we built a gamma-distribution, log-linked generalized linear mixed model (GLMM) with income group as the fixed effect, population as the random effect, and AFR as the response variable. To investigate the impact of income on IBI, we built another gamma-distribution, log-linked GLMM with income group and age as fixed effects, population as the random effect, and IBI as the response variable. Both GLMMs were evaluated for the effect of income group on each response variable (AFR and IBI) using a Type II ANOVA likelihood ratio test (*car* package) [83]. Post hoc pairwise comparisons adjusted with Tukey HSD (honestly significant difference) test were applied to test-paired income groups for AFR and IBI (*eemeans* package) [84]. To reduce computing time, we used a subsampling approach: each dataset was randomly sampled to *N* = 100,000 women for both datasets during statistical analysis only. We repeated this subsampling analysis 10 times, and results were similar for coefficient estimates (coefficients of variation of AFR model estimates: high-income = 0.0009, middle-income = 0.01, low-income = 0.01, natural fertility = 0.42, US WIC nonparticipants = 0.66, US WIC participant = 0.02; coefficients of variation of IBI model estimates: middle-income = 0.38, low-income = 0.02, natural fertility = 0.92, US WIC nonparticipants = 0.12, U.S. WIC participant = 0.09, age = 0.01) indicating subsampling produced repeatable results. The variation between subsamples did not change the results of the Type II ANOVA likelihood ratio test and pairwise comparisons. Summary statistics (mean, median, and mode) using full datasets were also computed (Tables 1 and 2). All data cleaning and analyses were conducted in R [85].

**Table 1:** Summary statistics for the AFR analysis

Income Group	Sample Size (*N*)	Mean AFR (years)	SE (years)	Modal AFR (years)
High-income	2,726,845	29.4	5.6	30
Middle-income	946,557	24.8	5.5	20
Low-income	147,700	19.3	3.5	19
Natural fertility	183	20.4	5.0	18
US WIC nonparticipant	802,084	28.5	5.4	29
US WIC participant	348,341	23.3	5.0	20

Income groups classified by country GNI per capita: low-income =  ≤ US $1085; middle-income = US $1086–13,205; high-income =  ≥ US $13,205

**Table 2:** Summary statistics for the IBI analysis

Income Group	Sample Size (*N*)	Mean IBI (months)	SE (months)	Modal IBI (months)
Middle-income	29,016	30.5	18.5	24
Low-income	33,949	27.0	13.0	26
Natural fertility	276	34.9	19.7	24
US WIC nonparticipant	438,441	39.8	24.3	21
US WIC participant	331,128	41.5	26.8	21

Income groups classified by country GNI per capita: low-income =  ≤ US $1085; middle-income = US $1086–13,205; high-income =  ≥ US $13,205

**Fig. 2:**
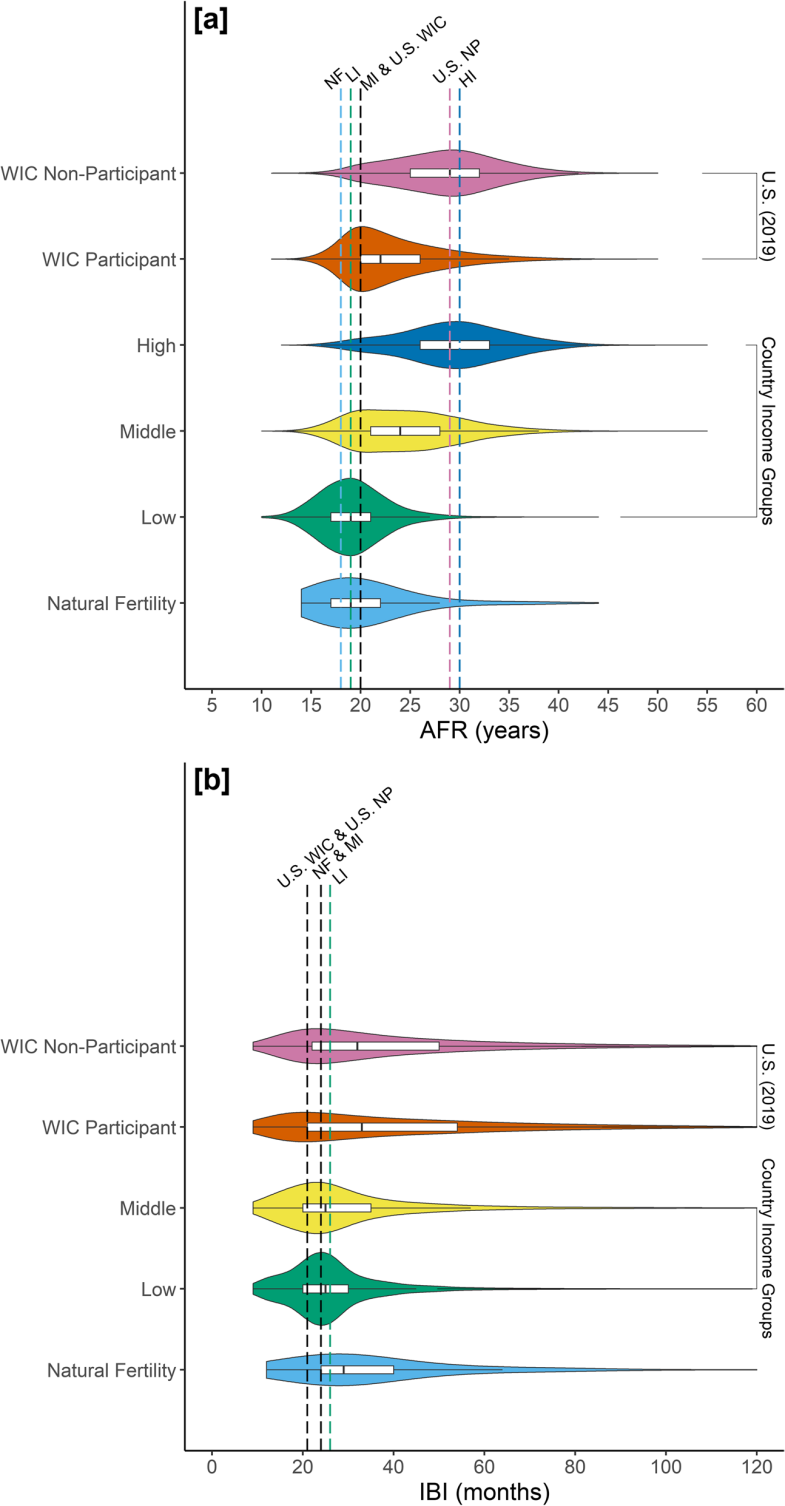
Distribution of **a** AFR (years) and **b** IBI (months) by income group. Vertical dashed lines indicate modal values for color-matched income groups. **a** Middle-income countries and WIC participants in the US have the same modal AFR value (20 years), represented by the black, dashed line. **b** WIC participants and nonparticipants in the US have the same modal IBI value (21 months), represented by the black, dashed line. The upper IBI range has been limited to 120 months. Abbreviations for modal lines: NF, natural fertility; LI, low-income; MI, middle-income; HI, high-income; US WIC, US WIC participant; US NP, US WIC nonparticipant

As expected, income group was a significant predictor of AFR (LRT: *χ*^2^ = 5989.5, *df* = 5, *p* < 0.001), with richer populations exhibiting a later AFR (Table 3). However, there was no difference in AFR between US WIC participants and women in natural fertility, low-income, or middle-income populations. The post hoc Tukey test revealed that only four income group pairwise comparisons for AFR were significant (*p* < 0.001): high-income was greater than low-income, middle-income, and natural fertility, and WIC nonparticipants were greater than participants. Modal AFR (i.e., the peak of the AFR distribution) among US WIC participants (20 years) is similar to lower income populations (low-income: 19 years, middle-income: 20 years, natural fertility: 18 years; Fig. 2a; Table 1).

**Table 3:** GLMM summaries for AFR and IBI as response variables

**Model 1.** Age at First Reproduction (AFR)		**Model 2.** Interbirth Interval (IBI)	
**Response variable**	*AFR (years)*	**Response variable**	*IBI (months)*
**Fixed effects**	*Estimate (SE)*	**Fixed effects**	*Estimate (SE)*
High-income (intercept)	3.36*** (0.02)	Low-income (intercept)	1.82*** (0.09)
Low-income	− 0.33*** (0.06)	Middle-income	0.11 (0.09)
Middle-income	− 0.29*** (0.02)	Natural fertility	0.15 (0.18)
Natural fertility	− 0.28** (0.11)	US WIC nonparticipants	0.24 (0.15)
US WIC nonparticipant	− 0.003 (0.11)	US WIC participants	0.32* (0.15)
US WIC participant	− 0.21 (0.11)	Age	0.06*** (0.0007)
**Random effects**	*SD*	**Random effects**	*SD*
Population (intercept)	0.02	Population (intercept)	0.08
Residual	0.19	Residual	0.58

^*^p < 0.05; **p < 0.01; ***p < 0.001

Income group was also a significant predictor of IBI, with richer populations having longer IBIs (LRT: *χ*^2^ = 490.6, *df* = 4, *p* < 0.001). The coefficient estimates for low-income, US WIC participants and age were significant (*p* < 0.05; Table 1). However, examining the distribution of IBI for each group, it is evident that women in industrialized and economically developed populations are capable of shorter IBI than women in less developed populations. Modal IBI (i.e., the peak of the IBI distribution) for women in the US (21 months) is shorter than in lower-income populations (low-income: 26 months; middle-income: 24 months; natural fertility: 24 months; Fig. 2b; Table 2). In contrast to the difference in AFR, WIC participants and nonparticipants in the US have the same modal IBI value (21 months; Fig. 2b; Table 2).

These comparisons demonstrate that most women in energy-rich, industrialized populations are capable of a greater reproductive output than energy-stressed populations, despite the well-established observation that women in industrialized populations often delay reproduction and reduce TFR via contraceptive methods [86]. Modal IBI values (the most common IBI in each group) were consistent with population-level energy status, with lower-income populations having longer modal IBI, as expected from an energy-stressed environment. Modal IBI for US women ages 20–29 is the shortest of all income groups. The lack of IBI difference between US WIC participants and nonparticipants may be explained by the prevalence of low cost and calorically dense ultra-processed foods that are readily available in the US [87, 88]. In addition, the WIC program subsidizes foods to increase affordability for pregnant women living in poverty [71, 72]. Poor nutritional content of low-cost food options notwithstanding, WIC participants may have sufficient caloric intake to shorten IBI comparable to nonparticipants.

## Reproduction in athletes

Athletes provide another avenue for investigating within-population variation in reproductive behavior and the role of energetics and energy status on human fertility. While often overlooked in anthropological investigations, athletes’ high levels of physical activity provide a useful point of comparison with subsistence populations. The limited studies conducted to date with pregnant elite athletes indicate that training volume decreases significantly over pregnancy, but that they maintain training volumes that are higher than sedentary counterparts and 2–3 times higher than standard guidelines for the third trimester [89–92]. Some athletes’ workloads exceed those in subsistence populations, making it easier to identify the effects of activity on reproduction.

Exercise, even at moderate levels without any change in body weight, can suppress reproductive hormones and increase the risk oligomenorrhea and amenorrhea [93, 94], characterized within a set of conditions known as Relative Energy Deficiency in Sport (RED-S) [95, 96]. Female athletes often exhibit delayed menarche, and the high training workloads typical among elite athletes can suppress ovulation entirely [94, 97]. These effects are consistent with the view that energy expended in physical activity trades off against energy available for reproductive function. While reproductive health and pregnancy outcomes for athletes are largely understudied, there is some evidence for reduced gestational weight gain and neonatal body size among athletes and nonathletes who exercise regularly [98–100].

## Future directions

The importance of energy in reproduction is well established, and decades of research among human populations worldwide have illuminated the connections between lifestyle, energy status, and fertility. Women with limited food availability, or with sizeable physical workloads, experience reproductive suppression and lower fertility. This response is likely adaptive, as pregnancy and lactation are energetically costly. Survival and future reproduction of both the mother and offspring could be compromised by a pregnancy initiated during a period of energy stress. Conversely, decreased workloads and improved food availability are associated with increased fertility.

While the details of human reproductive ecology have come into greater focus over the past several decades, questions remain. Energy status is not always clearly defined or operationalized, and the contributions of food availability on the one hand and physical activity workload on the other warrant further study. Measuring these variables can be challenging, particularly in field settings. Accelerometry is a useful tool for assessing physical activity, and can be improved with empirical tests of energy expenditure for common activities [5]. Doubly labeled water measures of energy expenditure, paired with measures of weight change, can be used to calculate energy intake [101].

Doubly labeled water measures of daily energy expenditure in a broader sample of farming and foraging societies would also improve our understanding of reproductive costs and energy budgets for subsistence populations. Studies from these settings have suggested that energy expenditures are often less than expected given the physically active lifestyle typical in small scale societies [28, 29, 102, 103]. However, few have used doubly labeled water to measure expenditures during pregnancy and lactation. A notable exception is a study by Heini and colleagues, which found that daily energy expenditures during late pregnancy did not differ from prepregnancy values among rural Gambian women [19]. Similarly, Pontzer and colleagues found no effect of pregnancy or lactation on daily energy expenditures in a small sample of women from the Hadza hunter-gatherer community [104]. These findings are consistent with the view that daily expenditure is constrained, with important implications for understanding energy allocation during reproduction, but additional work is needed to determine whether these results are robust and typical for women in subsistence populations.

Variation in fertility and reproductive behavior within large, diverse, industrialized populations also warrants further consideration. Much of the fertility research in industrialized countries is clinical in nature and focused on predicting and treating reproductive dysfunction or complications. These are important goals, but a more ecological and anthropological perspective may improve our understanding of the connections between lifestyle and reproductive outcomes. For example, while physical activity is known to improve cardiorespiratory and metabolic health, few studies have collected objective measures of physical activity alongside measures of energy expenditure during pregnancy or postpartum, and the role of physical activity in shaping energy allocation to reproduction remains understudied [98–100, 105]. Studies of pregnancy and postpartum energetics in athletes may be particularly fruitful, as their prodigious exercise workloads could provide a test of energetic limits hypotheses for human reproduction and enable researchers to identify energy trade-offs and other impacts of physical activity [34, 35].

## Supplementary Information

**Additional file 1:** **Table S1.** Countries included in the analysis (*n*=44). **Table S2.** Natural fertility (subsistence economy) populations included in analysis (*n*=2). 

## Data Availability

The datasets analyzed during the current study are subject to permission from organizations and parties who retain data rights. Details on accessing data are available in the supplementary materials. The R code is available in the OSF project repository, https://doi.org/10.17605/OSF.IO/JQZ2R.

## Abbreviations

AFR: Age at first reproduction
BMR: Basal metabolic rate
HPAA: Hypothalamic-pituitary-adrenal axis
IBI: Interbirth interval
TFR: Total fertility rate
WIC: Special Supplemental Nutrition Program for Women, Infants, and Children
GLMM: Generalized linear mixed model
GNI per capita: Gross National Income per capita, in US dollars
ANOVA: Analysis of variance
HSD: Honestly significant difference
RED-S: Relative energy deficiency in sport
US: United States of America
