# Supplementary material for: Impact of energy availability and physical activity on variation in fertility across human populations
Source: J Physiol Anthropol. 2023 Feb 24;42:1. doi: 10.1186/s40101-023-00318-3 (PMC9951524; doi:10.1186/s40101-023-00318-3)
Supplement: Supplementary file 1 — Additional file 1: Table S1. Countries included in the analysis (n=44). Table S2. Natural fertility (subsistence economy) populations included in analysis (n=2). [file 40101_2023_318_MOESM1_ESM.docx]

**SUPPLEMENTAL MATERIALS**

*Impact of energy availability and physical activity on variation in fertility across human populations*

**Table S1.** Countries included in the analysis (n=44).

| **Country** | **Continent** | **Income Group** |
| --- | --- | --- |
| South Africa | Africa | Middle-Income |
| Afghanistan | Asia | Low-Income |
| Armenia | Asia | Middle-Income |
| Azerbaijan | Asia | Middle-Income |
| Japan | Asia | High-Income |
| Kazakhstan | Asia | Middle-Income |
| Kyrgyz Republic | Asia | Middle-Income |
| Nepal | Asia | Middle-Income |
| Pakistan | Asia | Middle-Income |
| Philippines | Asia | Middle-Income |
| South Korea | Asia | High-Income |
| Taiwan | Asia | High-Income |
| Tajikistan | Asia | Low-Income |
| Uzbekistan | Asia | Middle-Income |
| Austria | Europe | High-Income |
| Belarus | Europe | Middle-Income |
| Bulgaria | Europe | Middle-Income |
| Croatia | Europe | High-Income |
| Czech Republic | Europe | High-Income |
| Denmark | Europe | High-Income |
| Estonia | Europe | High-Income |
| Finland | Europe | High-Income |
| Germany | Europe | High-Income |
| Hungary | Europe | High-Income |
| Iceland | Europe | High-Income |
| Italy | Europe | High-Income |
| Lithuania | Europe | High-Income |
| Moldova | Europe | Middle-Income |
| Netherlands | Europe | High-Income |
| Norway | Europe | High-Income |
| Poland | Europe | High-Income |
| Portugal | Europe | High-Income |
| Russia | Europe | Middle-Income |
| Slovakia | Europe | High-Income |
| Slovenia | Europe | High-Income |
| Spain | Europe | High-Income |
| Sweden | Europe | High-Income |
| Switzerland | Europe | High-Income |
| Ukraine | Europe | Middle-Income |
| United Kingdom | Europe | High-Income |
| Canada | North America | High-Income |
| United States | North America | High-Income |
| Chile | South America | High-Income |
| Peru | South America | Middle-Income |

**Table S2.** Natural fertility (subsistence economy) populations included in analysis (n=2).

| **Population** | **Continent** |
| --- | --- |
| Shuar | South America |
| Tsimane | South America |

**Data retrieval:**

*U.S. Dataset:* CDC National Center for Health Statistics: Vital Statistics Online Data Portal.

<https://www.cdc.gov/nchs/data_access/vitalstatsonline.htm>.

- Download U.S. birth data zipped folder for year 2019.
- Extract zipped folder.
- Move text file “Nat2019PublicUS.c20200506.r20200915.txt” to working directory.
- See associated user’s guide for year 2019 for data key and explanation.

*Tsimane Dataset:* Blackwell et al. (2015), Database S2.

<https://science.sciencemag.org/content/350/6263/970/tab-figures-data>.

- Download Database S2 under “Additional Files.”
- Move CSV file “aac7902-Blackwell-SM.database.S2.csv” to working directory.

*Shuar Dataset:* Madimenos et al. (2012), data access permission required.

Submit request at <https://www.shuarproject.org/data-sharing>.

- After receiving data file, convert Microsoft Excel spreadsheet to CSV file.
- Move CSV file to working directory.
- Update read.csv code in R script according to CSV file name.

*The Human Fertility Database:* Max Planck Institute for Demographic Research (MPIDR) & Vienna Institute of Demography (VID).

Free user account required, <https://www.humanfertility.org/cgi-bin/main.php>.

- Login to account.
- Click on “Data Availability” under the “Data” tab.
- Download “births” zip file under “Births” data type.
- Extract zipped folder.
- Move text file “birthsRRbo.txt” to working directory.

*The Demographic and Health Surveys (DHS) Program:* United States Agency for International Development (USAID), data access permission required.

Create account and submit request at <https://dhsprogram.com/data/new-user-registration.cfm>.

- Most recently available country datasets were used for this analysis. For some countries, this means survey years from the 1990s and 2000s, since surveys have not been conducted at these locations since.
- Request datasets for the following countries and years:
  - Afghanistan (2015)
  - Armenia (2015-2016)
  - Azerbaijan (2006)
  - Kazakhstan (1999)
  - Kyrgyz Republic (2012)
  - Moldova (2005)
  - Nepal (2016)
  - Pakistan (2017-2018)
  - Peru (2012)
  - Philippines (2017)
  - South Africa (2016)
  - Tajikistan (2017)
  - Ukraine (2007)
  - Uzbekistan (1996)
- Data type to be downloaded is “Standard DHS.” Save the SPSS “.SAV” file version for each country.
- Move files to working directory.

**R Code:**

(*R script is also available at OSF project repository*: <https://doi.org/10.17605/OSF.IO/JQZ2R>)

#Please see the Supplementary Materials for instructions on downloading the data

#Please see "Sadhir_AnalysisCountries.csv" and "Sadhir_NF.csv" files for the data used in the creation of Figure 1

#Set working directory to the file folder with stored data files

#Download packages with install.packages() for first-time use

#Load packages

library(car)

library(emmeans)

library(ggpubr)

library(ggtext)

library(haven)

library(jtools)

library(lme4)

library(modeest)

library(modEvA)

library(plyr)

library(rnaturalearth)

library(tidyverse)

#Increase the amount of memory allocated to R

memory.limit(size = 40000)

#### Data cleaning ####

##US Data

#Data source: CDC - 2019 birth data, https://www.cdc.gov/nchs/data_access/vitalstatsonline.htm

Births19 <- read.table("Nat2019PublicUS.c20200506.r20200915.txt", fill = T)

Births19.2 <- Births19 %>%

transform(Births19, V5 = ifelse(V5 == 1, V6, V5)) %>%

transform(Births19, V26 = ifelse(V26 < 0751, V26, V24)) %>%

transform(Births19, V26 = ifelse(V26 == "N10000000000001111N1", V25, V26)) %>%

transform(Births19, V26 = ifelse(V26 == "Y10000000000001111N1", V25, V26)) %>%

transform(Births19, V26 = ifelse(V26 == 9991, V25, V26)) %>%

filter(V26 > 3751) %>%

filter(V25 > 200) %>%

dplyr::select(V1, V11, V19, V17, V5, V25, V26, V27) %>%

mutate("BMI" = V26) %>%

dplyr::rename("Year" = V1, "Maternal Education" = V11, "IBI" = V19, "Live Births" = V17, "Age" = V5, "WIC" = V25, "Maternal Height" = V26, "Maternal Weight" = V27) %>%

mutate(`Maternal Education` = as.character(`Maternal Education`)) %>%

mutate(`Maternal Education` = str_sub(`Maternal Education`, -1)) %>%

mutate(IBI = as.character(IBI)) %>%

mutate(IBI = str_sub(IBI, end = -3)) %>%

mutate(IBI = as.numeric(IBI)) %>%

mutate(Age = str_sub(Age, end = 2)) %>%

mutate(WIC = str_sub(WIC, end = 1)) %>%

filter(IBI != "999" & IBI != "3" & IBI > 8 & `Live Births` < 30 & WIC != "2" & WIC != "3" & WIC != "4" & WIC != "5" & WIC != "6" & WIC != "7" & WIC != "9" & `Maternal Education` != "0") %>%

mutate(Population = c("US")) %>%

mutate(Group.Ed = case_when(endsWith(`Maternal Education`, "1") ~ "US.1", endsWith(`Maternal Education`, "2") ~ "US.2", endsWith(`Maternal Education`, "3") ~ "US.3", endsWith(`Maternal Education`, "4") ~ "US.4", endsWith(`Maternal Education`, "5") ~ "US.5", endsWith(`Maternal Education`, "6") ~ "US.6", endsWith(`Maternal Education`, "7") ~ "US.7", endsWith(`Maternal Education`, "8") ~ "US.8", endsWith(`Maternal Education`, "9") ~ "US.9", TRUE ~ NA_character_)) %>%

mutate(Group.WIC = case_when(endsWith(WIC, "Y") ~ "US.WIC", endsWith(WIC, "N") ~ "US.noWIC", endsWith(WIC, "U") ~ "US.unk", TRUE ~ NA_character_)) %>%

select(Population, Group.Ed, Group.WIC, `Age`, `Live Births`, IBI) %>%

dplyr::rename("Parity" = "Live Births") %>%

mutate(Age.Group = case_when(Age >= 45 & Age <= 50 ~ "45-50", Age >= 40 & Age <= 44 ~ "40-44", Age >= 35 & Age <= 39 ~ "35-39", Age >= 30 & Age <= 34 ~ "30-34", Age >= 25 & Age <= 29 ~ "25-29", Age >= 20 & Age <= 24 ~ "20-24", Age >= 15 & Age <= 19 ~ "15-19", Age >= 10 & Age <= 14 ~ "10-14")) %>%

mutate(Age = as.numeric(Age)) %>%

mutate(IBI = as.numeric(IBI)) %>%

mutate(Parity = as.numeric(Parity)) %>%

mutate(Group.Ed = case_when(Group.Ed == "US.1" ~ "1", Group.Ed == "US.2" ~ "2", Group.Ed == "US.3" ~ "3", Group.Ed == "US.4" ~ "3", Group.Ed == "US.5" ~ "3", Group.Ed == "US.6" ~ "3", Group.Ed == "US.7" ~ "3", Group.Ed == "US.8" ~ "3", Group.Ed == "US.9" ~ "NA", TRUE ~ NA_character_))

write_csv(Births19.2,"Births19.3.csv") #remove this for final R script

Births19.2 <- read_csv("Births19.3.csv")

US19.AFR <- Births19.2 %>% filter(IBI == 888)

US19.IBI <- Births19.2 %>% filter(IBI < 887)

##Tsimane data

#Data source: Blackwell et al. (2015) - Database S2, https://science.sciencemag.org/content/350/6263/970/tab-figures-data

Tsimane.Births <- read.csv("aac7902-Blackwell-SM.database.S2.csv")

Tsimane.AFR <- Tsimane.Births %>%

filter(Parity == 1) %>%

mutate(Population = c("Tsimane")) %>%

mutate(across(Age, round, 0)) %>%

mutate("IBI" = c(0)) %>%

select(Population, Age, Parity, IBI, Years.Education) %>%

mutate("Group.Ed" = Years.Education, "Group.WIC" = Population) %>%

mutate(Age.Group = case_when(Age >= 45 & Age <= 50 ~ "45-50", Age >= 40 & Age <= 44 ~ "40-44", Age >= 35 & Age <= 39 ~ "35-39", Age >= 30 & Age <= 34 ~ "30-34", Age >= 25 & Age <= 29 ~ "25-29", Age >= 20 & Age <= 24 ~ "20-24", Age >= 15 & Age <= 19 ~ "15-19", Age >= 10 & Age <= 14 ~ "10-14")) %>%

mutate(Age = as.numeric(Age)) %>%

mutate(IBI = as.numeric(IBI)) %>%

mutate(Parity = as.numeric(Parity)) %>%

select(Population, Age, Parity, IBI, Age.Group, Group.Ed, Group.WIC)

Tsimane.IBI <- Tsimane.Births %>%

filter(ConceptionEvent == 1) %>%

mutate(Population = c("Tsimane")) %>%

mutate(across(Age, round, 0)) %>%

mutate("IBI" = (Interval.End.Months + (272/(365/12)))) %>%

select(Population, Age, Parity, IBI, Years.Education) %>%

mutate("Group.Ed" = Years.Education, "Group.WIC" = Population) %>%

mutate(Age.Group = case_when(Age >= 45 & Age <= 50 ~ "45-50", Age >= 40 & Age <= 44 ~ "40-44", Age >= 35 & Age <= 39 ~ "35-39", Age >= 30 & Age <= 34 ~ "30-34", Age >= 25 & Age <= 29 ~ "25-29", Age >= 20 & Age <= 24 ~ "20-24", Age >= 15 & Age <= 19 ~ "15-19", Age >= 10 & Age <= 14 ~ "10-14")) %>%

mutate(Age = as.numeric(Age)) %>%

mutate(IBI = as.numeric(IBI)) %>%

mutate(Parity = as.numeric(Parity)) %>%

select(Population, Age, Parity, IBI, Age.Group, Group.Ed, Group.WIC)

##Shuar data

#Data source: Madimenos et al. (2012) - data access permission required, https://www.shuarproject.org/data-sharing

#Convert Excel spreadsheet to CSV file before loading data to R

Shuar.Births <- read.csv("Shuar Data 2011 - for Pontzer et al.csv") #Update CSV file name here according to the data file name received

Shuar.Births <- Shuar.Births %>%

pivot_longer(cols = Kid1Age:Kid15Age, names_to = c("KidOrder")) %>%

drop_na(value) %>%

mutate("KidAge" = value) %>%

mutate("Age2" = (Age - KidAge)) %>%

add_column("IBI2" = c(60, 96, 0, 60, 0, 48, 24, 24, 60, 60, 0, 12, 12, 12, 12, 24, 12, 24, 0, 0, 29.04, 0, 84, 48, 0, 12, 24, 36, 204, 24, 0, 24, 12, 36, 24, NA, 0, 36, 12, 36, 12, 15.6, 0, 36, NA, 48, 60, 72, 48, 24, 0, 12, 48, 48, 24, 24, 36, 0, 12, 12, 12, 12, 12, 12, 12, 84, 48, 12, 24, 12, 24, 12, 0, 36, 0, 24, 0, 40.8, 0, 36, 0, 12, 12, 36, 36, 0, 0, 24, 12, 24, 36, 24, 36, 24, 12, 12, 48, 0, 12, 24, 36, 36, 24, 12, 12, 24, NA, 12, 12, 24, 36, 12, 36, 24, 12, 48, 12, 12, 36, 0, 0, 24, 32.4, 0, 0, 12, 24, 23.5, 0, 24, 12, 42, 0, 24, 60, 15.6, 12,36, 0, 21.6, 0, 36, 24, 36, 44.4, 24, 0, 0, 48, 60, 0, 36, 36, 60, 0, 60, 96, 72, 12, 48, 36, 36, 24, 0, 0, 60, 36, 36, 12, 48, 31, 24, 0, 48, 48, NA, 12, 36, 24, 36, 48, 36, 24, 0, 24, 21, 0, 120, 60, 36, 23, 0, 40, 0, 0, 24, 60, 16, 0, 72, 48, NA, 0, 12, 0, 24, 24, 36, 32, 24, 82, 24, 0, 12, 0, 12, 24, 24, 12, 0, 18, 0, 36, 24, 24, 36, 24, 30, 12, 0, 24, 168, 0, 12, 72, 84, 0, 36, 12, 48, 24, 24, 48, 12, NA, 36, 0, 24, 0, 24, 12, 24, 12, 20.04, 0)) %>%

mutate(Population = c("Shuar")) %>%

mutate(across(Age2, round, 0)) %>%

mutate("Age" = Age2) %>%

mutate("Parity" = LiveBirthsTotal) %>%

mutate("IBI" = IBI2) %>%

select(Population, Age, Parity, IBI, KidOrder) %>%

mutate("Group.Ed" = Population, "Group.WIC" = Population) %>%

mutate(Age.Group = case_when(Age >= 45 & Age <= 50 ~ "45-50", Age >= 40 & Age <= 44 ~ "40-44", Age >= 35 & Age <= 39 ~ "35-39", Age >= 30 & Age <= 34 ~ "30-34", Age >= 25 & Age <= 29 ~ "25-29", Age >= 20 & Age <= 24 ~ "20-24", Age >= 15 & Age <= 19 ~ "15-19", Age >= 10 & Age <= 14 ~ "10-14")) %>%

mutate(Age = as.numeric(Age)) %>%

mutate(IBI = as.numeric(IBI)) %>%

mutate(Parity = as.numeric(Parity)) %>%

mutate(KidOrder = substr(KidOrder, 4, 4))

Shuar.AFR <- Shuar.Births %>%

select(Population, Age, Parity, IBI, Age.Group, Group.Ed, Group.WIC) %>%

filter(IBI == 0)

Shuar.IBI <- Shuar.Births %>%

select(Population, Age, Parity, IBI, Age.Group, Group.Ed, Group.WIC) %>%

filter(IBI > 0)

##The Human Fertility Database data

#Data source: Max Planck Institute for Demographic Research (MPIDR) & Vienna Institute of Demography (VID) - account required, https://www.humanfertility.org/cgi-bin/main.php

#From the downloaded folder, move only the text file "birthsRRbo.txt" to the working directory location

MP.Births <- read.table("birthsRRbo.txt", fill = T) %>%

slice(4:n()) %>%

select(1:3, 5) %>%

group_by(V1) %>%

filter(!V1 %in% c("DEUTE", "DEUTW", "GBR_NIR", "GBR_SCO", "GBRTENW", "UKR", "USA")) %>%

filter(V2 == max(V2)) %>%

mutate(V3 = replace(V3, V3 == "12-", "12")) %>%

mutate(V3 = replace(V3, V3 == "55+", "55")) %>%

mutate(V2 = as.numeric(V2), V3 = as.numeric(V3), V5 = as.numeric(V5)) %>%

dplyr::rename(country = V1, year = V2, age = V3, nbirths = V5) %>%

mutate(country = dplyr::recode(country, "AUT" = "Austria (2017)", "BLR" = "Belarus (2016)", "BGR" = "Bulgaria (2009)", "CAN" = "Canada (2016)", "CHE" = "Switzerland (2018)", "CHL" = "Chile (2005)", "CZE" = "Czech Republic (2018)", "DEUTNP" = "Germany (2017)", "DNK" = "Denmark (2019)", "ESP" = "Spain (2018)", "EST" = "Estonia (2017)", "FIN" = "Finland (2019)", "GBR_NP" = "UK (2018)", "HRV" = "Croatia (2017)", "HUN" = "Hungary (2017)", "ISL" = "Iceland (2018)", "ITA" = "Italy (2017)", "JPN" = "Japan (2018)", "KOR" = "South Korea (2018)", "LTU" = "Lithuania (2019)", "NLD" = "Netherlands (2018)", "NOR" = "Norway (2018)", "POL" = "Poland (2016)", "PRT" = "Portugal (2018)", "RUS" = "Russia (2018)", "SVK" = "Slovakia (2014)", "SVN" = "Slovenia (2017)", "SWE" = "Sweden (2019)", "TWN" = "Taiwan (2014)", "USA" = "US (2018)")) %>%

mutate(relbirths = nbirths/sum(nbirths))

MP.Births2 <- with(MP.Births, data.frame(Population = rep(country, nbirths), Group.Ed = rep(country, nbirths), Group.WIC = rep(country, nbirths), Age = rep(age, nbirths), Age.Group = rep(age, nbirths)))

MP.Births2 <- MP.Births2 %>% mutate(Age.Group = case_when(Age >= 45 & Age <= 50 ~ "45-50", Age >= 40 & Age <= 44 ~ "40-44", Age >= 35 & Age <= 39 ~ "35-39", Age >= 30 & Age <= 34 ~ "30-34", Age >= 25 & Age <= 29 ~ "25-29", Age >= 20 & Age <= 24 ~ "20-24", Age >= 15 & Age <= 19 ~ "15-19", Age >= 10 & Age <= 14 ~ "10-14")) %>%

mutate(IBI = NA) %>%

mutate(Age = as.numeric(Age)) %>%

mutate(IBI = as.numeric(IBI)) %>%

mutate(Parity = NA) %>%

mutate(Parity = as.numeric(Parity))

##USAID Demographic and Health Surveys Program

#Data source: United States Agency for International Development (USAID) - data access permission required, https://dhsprogram.com/data/new-user-registration.cfm

#Move SPSS .SAV file for each country to working directory location

Afghanistan15 <- read_sav("AFBR71FL.SAV")

Armenia1516 <- read_sav("AMBR72FL.SAV")

Azerbaijan06 <- read_sav("AZBR52FL.SAV")

Kazakhstan99 <- read_sav("KKBR42FL.SAV")

KyrgyzRepublic12 <- read_sav("KYBR61FL.SAV")

Moldova05 <- read_sav("MBBR53FL.SAV")

Nepal16 <- read_sav("NPBR7HFL.SAV")

Pakistan1718 <- read_sav("PKBR71FL.SAV")

Peru12 <- read_sav("PEBR6IFL.SAV")

Philippines17 <- read_sav("PHBR71FL.SAV")

SouthAfrica16 <- read_sav("ZABR71FL.SAV")

Tajikistan17 <- read_sav("TJBR71FL.SAV")

Ukraine07 <- read_sav("UABR51FL.SAV")

Uzbekistan96 <- read_sav("UZBR31FL.SAV")

Pop.list <- list(Afghanistan15, Armenia1516, Azerbaijan06, Kazakhstan99, KyrgyzRepublic12, Moldova05, Nepal16, Pakistan1718, Peru12, Philippines17, SouthAfrica16, Tajikistan17, Ukraine07, Uzbekistan96)

Pop.names <- c("Afghanistan15", "Armenia1516", "Azerbaijan06", "Kazakhstan99", "KyrgyzRepublic12", "Moldova05", "Nepal16", "Pakistan1718", "Peru12", "Philippines17", "SouthAfrica16", "Tajikistan17", "Ukraine07", "Uzbekistan96")

#Create empty list and data frame to prepare for loop

USAIDpop <- list()

#Loop

for(i in 1:length(Pop.list)){

USAIDpop[[i]] <- data.frame("Year of Interview" = Pop.list[[i]]$V007, "Date of Birth: CMC" = Pop.list[[i]]$V011, "Age" = Pop.list[[i]]$V012, "Total Children Born" = Pop.list[[i]]$V201, "Sons at Home" = Pop.list[[i]]$V202, "Daughters at Home" = Pop.list[[i]]$V203, "Sons Elsewhere" = Pop.list[[i]]$V204, "Daughters Elsewhere" = Pop.list[[i]]$V205, "Sons Dead" = Pop.list[[i]]$V206, "Daughters Dead" = Pop.list[[i]]$V207, "Date of First Birth: CMC" = Pop.list[[i]]$V211, "Age at First Birth" = Pop.list[[i]]$V212, "Number of Living Children" = Pop.list[[i]]$V218, "Weight" = Pop.list[[i]]$V437, "Height" = Pop.list[[i]]$V438, "BMI" = Pop.list[[i]]$V445, "Age of Child" = Pop.list[[i]]$B8, "Preceding Birth Interval" = Pop.list[[i]]$B11, "Succeeding Birth Interval" = Pop.list[[i]]$B12, "Highest Education Level" = Pop.list[[i]]$V106, "Highest Year of Education" = Pop.list[[i]]$V107)

USAIDpop[[i]]$Population <- Pop.names[i]

}

Afghanistan15 <- matrix(unlist(USAIDpop[[1]]), ncol = 22)

Armenia1516 <- matrix(unlist(USAIDpop[[2]]), ncol = 22)

Azerbaijan06 <- matrix(unlist(USAIDpop[[3]]), ncol = 22)

Kazakhstan99 <- matrix(unlist(USAIDpop[[4]]), ncol = 22)

KyrgyzRepublic12 <- matrix(unlist(USAIDpop[[5]]), ncol = 22)

Moldova05 <- matrix(unlist(USAIDpop[[6]]), ncol = 22)

Nepal16 <- matrix(unlist(USAIDpop[[7]]), ncol = 22)

Pakistan1718 <- matrix(unlist(USAIDpop[[8]]), ncol = 22)

Peru12 <- matrix(unlist(USAIDpop[[9]]), ncol = 22)

Philippines17 <- matrix(unlist(USAIDpop[[10]]), ncol = 22)

SouthAfrica16 <- matrix(unlist(USAIDpop[[11]]), ncol = 22)

Tajikistan17 <- matrix(unlist(USAIDpop[[12]]), ncol = 22)

Ukraine07 <- matrix(unlist(USAIDpop[[13]]), ncol = 22)

Uzbekistan96 <- matrix(unlist(USAIDpop[[14]]), ncol = 22)

#Bind all unlisted USAID populations

USAIDpop <- data.frame(rbind(Afghanistan15, Armenia1516, Azerbaijan06, Kazakhstan99, KyrgyzRepublic12, Moldova05, Nepal16, Pakistan1718, Peru12, Philippines17, SouthAfrica16, Tajikistan17, Ukraine07, Uzbekistan96))

write.csv(USAIDpop,"USAIDpop.csv", row.names = FALSE) #remove this for final R script

USAIDpop <- read_csv("USAIDpop.csv")

#New data frame for AFR with selected and renamed variables

USAIDpop.AFR <- USAIDpop %>% select(X22, X12, X4, X18, X20) %>%

dplyr::rename("Population" = X22, "Age" = X12, "Parity" = X4, "IBI" = X18, "Group.Ed" = X20) %>%

mutate("Group.WIC" = Population) %>%

mutate(Age.Group = case_when(Age >= 45 & Age <= 50 ~ "45-50", Age >= 40 & Age <= 44 ~ "40-44", Age >= 35 & Age <= 39 ~ "35-39", Age >= 30 & Age <= 34 ~ "30-34", Age >= 25 & Age <= 29 ~ "25-29", Age >= 20 & Age <= 24 ~ "20-24", Age >= 15 & Age <= 19 ~ "15-19", Age >= 10 & Age <= 14 ~ "10-14")) %>%

mutate(Age = as.numeric(Age)) %>%

mutate(IBI = as.numeric(IBI)) %>%

mutate(Parity = as.numeric(Parity))

#New data frame for IBI with selected and renamed variables

USAIDpop.IBI <- USAIDpop %>% select(X22, X4, X18, X3, X20) %>%

dplyr::rename("Population" = X22, "Age" = X3, "Parity" = X4, "IBI" = X18, "Group.Ed" = X20) %>%

mutate("Group.WIC" = Population) %>%

mutate(Age.Group = case_when(Age >= 45 & Age <= 50 ~ "45-50", Age >= 40 & Age <= 44 ~ "40-44", Age >= 35 & Age <= 39 ~ "35-39", Age >= 30 & Age <= 34 ~ "30-34", Age >= 25 & Age <= 29 ~ "25-29", Age >= 20 & Age <= 24 ~ "20-24", Age >= 15 & Age <= 19 ~ "15-19", Age >= 10 & Age <= 14 ~ "10-14")) %>%

mutate(Age = as.numeric(Age)) %>%

mutate(IBI = as.numeric(IBI)) %>%

mutate(Parity = as.numeric(Parity))

#### Creating final datasets ####

AFRsample <- rbind(USAIDpop.AFR, US19.AFR, Tsimane.AFR, Shuar.AFR) %>%

mutate(Population = dplyr::recode(Population, "Afghanistan15" = "Afghanistan (2015)", "Armenia1516" = "Armenia (2015-2016)", "Azerbaijan06" = "Azerbaijan (2006)", "Kazakhstan99" = "Kazakhstan (1999)", "KyrgyzRepublic12" = "Kyrgyz Republic (2012)", "Moldova05" = "Moldova (2005)", "Nepal16" = "Nepal (2016)", "Pakistan1718" = "Pakistan (2017-2018)", "Peru12" = "Peru (2012)", "Philippines17" = "Philippines (2017)", "Shuar" = "Shuar", "SouthAfrica16" = "South Africa (2016)", "Tajikistan17" = "Tajikistan (2017)", "Tsimane" = "Tsimane", "Ukraine07" = "Ukraine (2007)", "US" = "US (2019)", "Uzbekistan96" = "Uzbekistan (1996)")) %>%

mutate(Income = case_when(

Population == "Afghanistan (2015)" ~ "Low",

Population == "Armenia (2015-2016)" ~ "Middle",

Population == "Azerbaijan (2006)" ~ "Middle",

Population == "Kazakhstan (1999)" ~ "Middle",

Population == "Kyrgyz Republic (2012)" ~ "Middle",

Population == "Moldova (2005)" ~ "Middle",

Population == "Nepal (2016)" ~ "Middle",

Population == "Pakistan (2017-2018)" ~ "Middle",

Population == "Peru (2012)" ~ "Middle",

Population == "Philippines (2017)" ~ "Middle",

Population == "Shuar" ~ "Natural Fertility",

Population == "South Africa (2016)" ~ "Middle",

Population == "Tajikistan (2017)" ~ "Low",

Population == "Tsimane" ~ "Natural Fertility",

Population == "Ukraine (2007)" ~ "Middle",

Population == "US (2019)" ~ "High",

Population == "Uzbekistan (1996)" ~ "Middle",

Population == "Austria (2017)" ~ "High",

Population == "Belarus (2016)" ~ "Middle",

Population == "Bulgaria (2009)" ~ "Middle",

Population == "Canada (2016)" ~ "High",

Population == "Switzerland (2018)" ~ "High",

Population == "Chile (2005)" ~ "High",

Population == "Czech Republic (2018)" ~ "High",

Population == "Germany (2017)" ~ "High",

Population == "Denmark (2019)" ~ "High",

Population == "Spain (2018)" ~ "High",

Population == "Estonia (2017)" ~ "High",

Population == "Finland (2019)" ~ "High",

Population == "UK (2018)" ~ "High",

Population == "Croatia (2017)" ~ "High",

Population == "Hungary (2017)" ~ "High",

Population == "Iceland (2018)" ~ "High",

Population == "Italy (2017)" ~ "High",

Population == "Japan (2018)" ~ "High",

Population == "South Korea (2018)" ~ "High",

Population == "Lithuania (2019)" ~ "High",

Population == "Netherlands (2018)" ~ "High",

Population == "Norway (2018)" ~ "High",

Population == "Poland (2016)" ~ "High",

Population == "Portugal (2018)" ~ "High",

Population == "Russia (2018)" ~ "Middle",

Population == "Slovakia (2014)" ~ "High",

Population == "Slovenia (2017)" ~ "High",

Population == "Sweden (2019)" ~ "High",

Population == "Taiwan (2014)" ~ "High")) %>%

filter(Age > 9) %>%

filter(!(Group.WIC == "US.unk")) %>%

mutate(Income = dplyr::recode(Income, "WIC" = "WIC Participant", "No WIC" = "WIC Non-Participant"))

USAIDpop.IBI <- USAIDpop.IBI %>% mutate(Group.Ed = as.numeric(Group.Ed))

US19.IBI <- US19.IBI %>% mutate(Group.Ed = as.numeric(Group.Ed))

Tsimane.IBI <- Tsimane.IBI %>% mutate(Group.Ed = as.numeric(Group.Ed))

Shuar.IBI <- Shuar.IBI %>% mutate(Group.Ed = as.numeric(Group.Ed))

IBIsample <- bind_rows(USAIDpop.IBI, US19.IBI, Tsimane.IBI, Shuar.IBI) %>%

mutate(Population = dplyr::recode(Population, "Afghanistan15" = "Afghanistan (2015)", "Armenia1516" = "Armenia (2015-2016)", "Azerbaijan06" = "Azerbaijan (2006)", "Kazakhstan99" = "Kazakhstan (1999)", "KyrgyzRepublic12" = "Kyrgyz Republic (2012)", "Moldova05" = "Moldova (2005)", "Nepal16" = "Nepal (2016)", "Pakistan1718" = "Pakistan (2017-2018)", "Peru12" = "Peru (2012)", "Philippines17" = "Philippines (2017)", "Shuar" = "Shuar", "SouthAfrica16" = "South Africa (2016)", "Tajikistan17" = "Tajikistan (2017)", "Tsimane" = "Tsimane", "Ukraine07" = "Ukraine (2007)", "US" = "US (2019)", "Uzbekistan96" = "Uzbekistan (1996)")) %>%

mutate(Income = case_when(

Population == "Afghanistan (2015)" ~ "Low",

Population == "Armenia (2015-2016)" ~ "Middle",

Population == "Azerbaijan (2006)" ~ "Middle",

Population == "Kazakhstan (1999)" ~ "Middle",

Population == "Kyrgyz Republic (2012)" ~ "Middle",

Population == "Moldova (2005)" ~ "Middle",

Population == "Nepal (2016)" ~ "Middle",

Population == "Pakistan (2017-2018)" ~ "Middle",

Population == "Peru (2012)" ~ "Middle",

Population == "Philippines (2017)" ~ "Middle",

Population == "Shuar" ~ "Natural Fertility",

Population == "South Africa (2016)" ~ "Middle",

Population == "Tajikistan (2017)" ~ "Low",

Population == "Tsimane" ~ "Natural Fertility",

Population == "Ukraine (2007)" ~ "Middle",

Population == "US (2019)" ~ "High",

Population == "Uzbekistan (1996)" ~ "Middle")) %>%

filter(Age > 19 & Age < 30) %>%

filter(IBI > 8) %>%

drop_na(IBI) %>%

mutate(across(IBI, round, 0)) %>%

filter(!(Group.WIC == "US.unk")) %>%

mutate(Income = dplyr::recode(Income, "WIC" = "WIC Participant", "No WIC" = "WIC Non-Participant"))

#### Summary tables ####

AFRsummary <- AFRsample %>%

group_by(Income) %>%

summarize(count = n(), mean(Age, na_rm = T), sd(Age, na.rm = T), mfv(Age, na_rm = T)) %>%

mutate(across("mean(Age, na_rm = T)", round, 1)) %>%

mutate(across("sd(Age, na.rm = T)", round, 1)) %>%

rename("Income Group" = Income, "Sample Size" = count, "Mean AFR (years)" = "mean(Age, na_rm = T)", "SD (years)" = "sd(Age, na.rm = T)", "Modal AFR (years)" = "mfv(Age, na_rm = T)")

IBIsummary <- IBIsample %>%

group_by(Income) %>%

summarize(count = n(), mean(IBI, na_rm = T), sd(IBI, na.rm = T), mfv(IBI, na_rm = T)) %>%

mutate(across("mean(IBI, na_rm = T)", round, 1)) %>%

mutate(across("sd(IBI, na.rm = T)", round, 1)) %>%

rename("Income Group" = Income, "Sample Size" = count, "Mean IBI (months)" = "mean(IBI, na_rm = T)", "SD (months)" = "sd(IBI, na.rm = T)", "Modal IBI (months)" = "mfv(IBI, na_rm = T)")

#### Generalized linear mixed models (GLMMs) ####

##AFR model with resampling (10 times)

#Create empty lists to prepare for loop

AFR.subsample <- list()

AFRmod <- list()

#Loop

for(i in 1:10){

AFR.subsample[[i]] <- AFRsample[sample(nrow(AFRsample), 100000), ] #Random sample of the total AFR sample taken to compute GLMM on a consumer-model computer (16 GB RAM)

AFRmod[[i]] <- glmer(Age ~ Income + Group.Ed + (1 | Population), data = AFR.subsample[[i]], family = Gamma(link = "log"))

}

#Model diagnostics of first subsampled dataset

summary(AFRmod[[1]])

summ(AFRmod[[1]])

car::Anova(AFRmod[[1]]) #Chisq=5989.5; Df=5; p<0.001 (p<2.2e-16); results will vary slightly based on random sample

#Pairwise comparisons adjusted with Tukey HSD

emmeans(AFRmod[[1]], pairwise ~ Group.Ed, adjust = "tukey")

#Calculate the coefficient of variation (CV) for coefficient estimates of the 10 subsampled datasets

AFRsumm1 <- summ(AFRmod[[1]])

AFRsumm1 <- data.frame(unlist(AFRsumm1[["coeftable"]])) %>%

mutate(model = c("mod1"))

AFRsumm2 <- summ(AFRmod[[2]])

AFRsumm2 <- data.frame(unlist(AFRsumm2[["coeftable"]])) %>%

mutate(model = c("mod2"))

AFRsumm3 <- summ(AFRmod[[3]])

AFRsumm3 <- data.frame(unlist(AFRsumm3[["coeftable"]])) %>%

mutate(model = c("mod3"))

AFRsumm4 <- summ(AFRmod[[4]])

AFRsumm4 <- data.frame(unlist(AFRsumm4[["coeftable"]])) %>%

mutate(model = c("mod4"))

AFRsumm5 <- summ(AFRmod[[5]])

AFRsumm5 <- data.frame(unlist(AFRsumm5[["coeftable"]])) %>%

mutate(model = c("mod5"))

AFRsumm6 <- summ(AFRmod[[6]])

AFRsumm6 <- data.frame(unlist(AFRsumm6[["coeftable"]])) %>%

mutate(model = c("mod6"))

AFRsumm7 <- summ(AFRmod[[7]])

AFRsumm7 <- data.frame(unlist(AFRsumm7[["coeftable"]])) %>%

mutate(model = c("mod7"))

AFRsumm8 <- summ(AFRmod[[8]])

AFRsumm8 <- data.frame(unlist(AFRsumm8[["coeftable"]])) %>%

mutate(model = c("mod8"))

AFRsumm9 <- summ(AFRmod[[9]])

AFRsumm9 <- data.frame(unlist(AFRsumm9[["coeftable"]])) %>%

mutate(model = c("mod9"))

AFRsumm10 <- summ(AFRmod[[10]])

AFRsumm10 <- data.frame(unlist(AFRsumm10[["coeftable"]])) %>%

mutate(model = c("mod10"))

AFR.CV <- rbind(AFRsumm1, AFRsumm2, AFRsumm3, AFRsumm4, AFRsumm5, AFRsumm6, AFRsumm7, AFRsumm8, AFRsumm9, AFRsumm10)

AFR.CV.HI <- AFR.CV[c("(Intercept)", "(Intercept)1", "(Intercept)2", "(Intercept)3", "(Intercept)4", "(Intercept)5", "(Intercept)6", "(Intercept)7", "(Intercept)8", "(Intercept)9"), ]

sd(AFR.CV.HI$Est.) / mean(AFR.CV.HI$Est.) #CV = 0.0009

AFR.CV.LI <- AFR.CV[c("IncomeLow", "IncomeLow1", "IncomeLow2", "IncomeLow3", "IncomeLow4", "IncomeLow5", "IncomeLow6", "IncomeLow7", "IncomeLow8", "IncomeLow9"), ]

sd(AFR.CV.LI$Est.) / mean(AFR.CV.LI$Est.) #CV (absolute value) = 0.0134

AFR.CV.MI <- AFR.CV[c("IncomeMiddle", "IncomeMiddle1", "IncomeMiddle2", "IncomeMiddle3", "IncomeMiddle4", "IncomeMiddle5", "IncomeMiddle6", "IncomeMiddle7", "IncomeMiddle8", "IncomeMiddle9"), ]

sd(AFR.CV.MI$Est.) / mean(AFR.CV.MI$Est.) #CV (absolute value) = 0.0129

AFR.CV.NF <- AFR.CV[c("IncomeNatural Fertility", "IncomeNatural Fertility1", "IncomeNatural Fertility2", "IncomeNatural Fertility3", "IncomeNatural Fertility4", "IncomeNatural Fertility5", "IncomeNatural Fertility6", "IncomeNatural Fertility7", "IncomeNatural Fertility8", "IncomeNatural Fertility9"), ]

sd(AFR.CV.NF$Est.) / mean(AFR.CV.NF$Est.) #CV (absolute value) = 0.4209

AFR.CV.NP <- AFR.CV[c("IncomeWIC Non-Participant", "IncomeWIC Non-Participant1", "IncomeWIC Non-Participant2", "IncomeWIC Non-Participant3", "IncomeWIC Non-Participant4", "IncomeWIC Non-Participant5", "IncomeWIC Non-Participant6", "IncomeWIC Non-Participant7", "IncomeWIC Non-Participant8", "IncomeWIC Non-Participant9"), ]

sd(AFR.CV.NP$Est.) / mean(AFR.CV.NP$Est.) #CV (absolute value) = 0.6601

AFR.CV.WIC <- AFR.CV[c("IncomeWIC Participant", "IncomeWIC Participant1", "IncomeWIC Participant2", "IncomeWIC Participant3", "IncomeWIC Participant4", "IncomeWIC Participant5", "IncomeWIC Participant6", "IncomeWIC Participant7", "IncomeWIC Participant8", "IncomeWIC Participant9"), ]

sd(AFR.CV.WIC$Est.) / mean(AFR.CV.WIC$Est.) #CV (absolute value) = 0.0221

#IBI model with resampling (10 times)

#Create empty lists to prepare for loop

IBI.subsample <- list()

IBImod <- list()

#Loop

for(i in 1:10){

IBI.subsample[[i]] <- IBIsample[sample(nrow(IBIsample), 1000), ] #Random sample of the total IBI sample taken to compute GLMM on a consumer-model computer (16 GB RAM)

IBImod[[i]] <- glmer(IBI ~ Income + Age + Group.Ed + (1 | Population), data = IBI.subsample[[i]], family = Gamma(link = "log"))

}

#Model diagnostics of first subsampled dataset

summary(IBImod[[1]])

summ(IBImod[[1]])

Anova(IBImod[[1]]) #Chisq = 490.56; Df=2; p<0.001 (p<2.2e-16); results will vary slightly based on random sample

#Pairwise comparisons adjusted with Tukey HSD

emmeans(IBImod[[4]], pairwise ~ Income, adjust = "tukey")

#Calculate the coefficient of variation (CV) for coefficient estimates of the 10 subsampled datasets

IBIsumm1 <- summ(IBImod[[1]])

IBIsumm1 <- data.frame(unlist(IBIsumm1[["coeftable"]])) %>%

mutate(model = c("mod1"))

IBIsumm2 <- summ(IBImod[[2]])

IBIsumm2 <- data.frame(unlist(IBIsumm2[["coeftable"]])) %>%

mutate(model = c("mod2"))

IBIsumm3 <- summ(IBImod[[3]])

IBIsumm3 <- data.frame(unlist(IBIsumm3[["coeftable"]])) %>%

mutate(model = c("mod3"))

IBIsumm4 <- summ(IBImod[[4]])

IBIsumm4 <- data.frame(unlist(IBIsumm4[["coeftable"]])) %>%

mutate(model = c("mod4"))

IBIsumm5 <- summ(IBImod[[5]])

IBIsumm5 <- data.frame(unlist(IBIsumm5[["coeftable"]])) %>%

mutate(model = c("mod5"))

IBIsumm6 <- summ(IBImod[[6]])

IBIsumm6 <- data.frame(unlist(IBIsumm6[["coeftable"]])) %>%

mutate(model = c("mod6"))

IBIsumm7 <- summ(IBImod[[7]])

IBIsumm7 <- data.frame(unlist(IBIsumm7[["coeftable"]])) %>%

mutate(model = c("mod7"))

IBIsumm8 <- summ(IBImod[[8]])

IBIsumm8 <- data.frame(unlist(IBIsumm8[["coeftable"]])) %>%

mutate(model = c("mod8"))

IBIsumm9 <- summ(IBImod[[9]])

IBIsumm9 <- data.frame(unlist(IBIsumm9[["coeftable"]])) %>%

mutate(model = c("mod9"))

IBIsumm10 <- summ(IBImod[[10]])

IBIsumm10 <- data.frame(unlist(IBIsumm10[["coeftable"]])) %>%

mutate(model = c("mod10"))

IBI.CV <- rbind(IBIsumm1, IBIsumm2, IBIsumm3, IBIsumm4, IBIsumm5, IBIsumm6, IBIsumm7, IBIsumm8, IBIsumm9, IBIsumm10)

IBI.CV.LI <- IBI.CV[c("(Intercept)", "(Intercept)1", "(Intercept)2", "(Intercept)3", "(Intercept)4", "(Intercept)5", "(Intercept)6", "(Intercept)7", "(Intercept)8", "(Intercept)9"), ]

sd(IBI.CV.LI$Est.) / mean(IBI.CV.LI$Est.) #CV = 0.0177

IBI.CV.MI <- IBI.CV[c("IncomeMiddle", "IncomeMiddle1", "IncomeMiddle2", "IncomeMiddle3", "IncomeMiddle4", "IncomeMiddle5", "IncomeMiddle6", "IncomeMiddle7", "IncomeMiddle8", "IncomeMiddle9"), ]

sd(IBI.CV.MI$Est.) / mean(IBI.CV.MI$Est.) #CV = 0.3839

IBI.CV.NF <- IBI.CV[c("IncomeNatural Fertility", "IncomeNatural Fertility1", "IncomeNatural Fertility2", "IncomeNatural Fertility3", "IncomeNatural Fertility4", "IncomeNatural Fertility5", "IncomeNatural Fertility6", "IncomeNatural Fertility7", "IncomeNatural Fertility8", "IncomeNatural Fertility9"), ]

sd(IBI.CV.NF$Est.) / mean(IBI.CV.NF$Est.) #CV = 0.9228

IBI.CV.NP <- IBI.CV[c("IncomeWIC Non-Participant", "IncomeWIC Non-Participant1", "IncomeWIC Non-Participant2", "IncomeWIC Non-Participant3", "IncomeWIC Non-Participant4", "IncomeWIC Non-Participant5", "IncomeWIC Non-Participant6", "IncomeWIC Non-Participant7", "IncomeWIC Non-Participant8", "IncomeWIC Non-Participant9"), ]

sd(IBI.CV.NP$Est.) / mean(IBI.CV.NP$Est.) #CV = 0.1211

IBI.CV.WIC <- IBI.CV[c("IncomeWIC Participant", "IncomeWIC Participant1", "IncomeWIC Participant2", "IncomeWIC Participant3", "IncomeWIC Participant4", "IncomeWIC Participant5", "IncomeWIC Participant6", "IncomeWIC Participant7", "IncomeWIC Participant8", "IncomeWIC Participant9"), ]

sd(IBI.CV.WIC$Est.) / mean(IBI.CV.WIC$Est.) #CV = 0.0871

IBI.CV.age <- IBI.CV[c("Age", "Age1", "Age2", "Age3", "Age4", "Age5", "Age6", "Age7", "Age8", "Age9"), ]

sd(IBI.CV.age$Est.) / mean(IBI.CV.age$Est.) #CV = 0.0102

#### Figure 1 ####

#Load .csv file for countries used in the analysis (status = 1)

analysis.countries <- read_csv("Sadhir_AnalysisCountries.csv")

#Load all countries with associated GPS coordinates

country.distr <- ne_countries(scale = "medium", returnclass = "sf") %>%

select(name, pop_est, gdp_md_est, continent, geometry)

#Merge datasets of all countries with countries used in the analysis

country.dataset <- merge(country.distr, analysis.countries, by = "name", all.x = TRUE)

#Replace status = NA with 0, denoting that the country was not used in the analysis

country.dataset <- country.dataset %>% replace_na(list(status = 0))

#Load .csv file for natural fertility populations with associated approximate GPS coordinates

NF.dataset <- read_csv("Sadhir_NF.csv")

#World map with shaded countries and labeled natural fertility populations used in the analysis

ggplot() +

geom_sf(data = country.dataset, aes(fill = factor(status))) +

scale_fill_manual(values=c("#F3F8F2", "#71A7C6")) +

geom_point(data = NF.dataset, aes(x = lon, y = lat), size = 3, shape = 23, fill = "#A24936") +

geom_text(data = NF.dataset, aes(x = lon, y = lat, label = name), hjust = 1, nudge_x = -5, size = 4) +

theme_classic() +

theme(legend.position = "none", axis.title.x = element_blank(),

axis.title.y = element_blank())

#Save Figure 1

ggsave("studymap.tiff", plot = last_plot(), device = tiff(), dpi = 400, width = 12, height = 6)

#### Figure 2 ####

#Load colorblind-friendly palettes

cbPalette <- c("#56B4E9", "#009E73", "#F0E442", "#0072B2", "#D55E00", "#CC79A7")

cbPalette2 <- c("#56B4E9", "#009E73", "#F0E442", "#D55E00", "#CC79A7")

#Violin plots of AFR data (total sample) with overlayed boxplots and modal value lines

LineType <- c("Natural Fertility" = "dashed", "Low Income" = "F1", "Middle Income & U.S. WIC Participants" = "dotted", "U.S. WIC Non-Participants" = "longdash", "High Income"= "dotdash")

AFRplot <- ggplot() +

geom_violin(data = AFRsample %>% filter(Age > 9) %>% mutate(Income = factor(Income, levels = c("Natural Fertility", "Low", "Middle", "High", "WIC Participant", "WIC Non-Participant"))), aes(x = Income, y = Age, fill = Income), adjust = 3) +

geom_boxplot(data = AFRsample %>% filter(Age > 9) %>% mutate(Income = factor(Income, levels = c("Natural Fertility", "Low", "Middle", "High", "WIC Participant", "WIC Non-Participant"))), aes(x = Income, y = Age), outlier.shape = NA, width = 0.1) +

xlab("") + ylab("AFR (years)") +

theme_classic(base_size = 20) +

theme(text = element_text(size = 25)) +

geom_bracket(xmin = "Low", xmax = "High", y.position = 60, label = "Country Income Groups", tip.length = c(0.25, 0.02), label.size = 6.5, coord.flip = TRUE) +

geom_bracket(xmin = "WIC Participant", xmax = "WIC Non-Participant", label.size = 6.5, y.position = 60, label = "U.S. (2019)", tip.length = c(0.1, 0.1), coord.flip = TRUE) +

scale_y_continuous(limits = c(5, 60), breaks = c(5, 10, 15, 20, 25, 30, 35, 40, 45, 50, 55, 60)) +

geom_segment(mapping = aes(x = 0, xend = 7, y = 18, yend = 18), linetype = "longdash", size = 0.9, color = "#56B4E9") +

geom_segment(mapping = aes(x = 0, xend = 7, y = 19, yend = 19), linetype = "longdash", size = 0.9, color = "#009E73") +

geom_segment(mapping = aes(x = 0, xend = 7, y = 20, yend = 20), linetype = "longdash", size = 0.9, color = "black") +

geom_segment(mapping = aes(x = 0, xend = 7, y = 29, yend = 29), linetype = "longdash", size = 0.9, color = "#CC79A7") +

geom_segment(mapping = aes(x = 0, xend = 7, y = 30, yend = 30), linetype = "longdash", size = 0.9, color = "#0072B2") +

theme(legend.position = "none") +

scale_fill_manual(values = cbPalette) +

annotate(geom = "text", label = "NF", x = 7.1, y = 17.4, angle = 40, size = 6) +

annotate(geom = "text", label = "LI", x = 7.15, y = 19.2, angle = 40, size = 6) +

annotate(geom = "text", label = "MI & U.S. WIC", x = 7.4, y = 24.3, angle = 40, size = 6) +

annotate(geom = "text", label = "U.S. NP", x = 7.3, y = 29.8, angle = 40, size = 6) +

annotate(geom = "text", label = "HI", x = 7.1, y = 30.8, angle = 40, size = 6) +

annotate(geom = "text", label = "[a]", x = 7.8, y = 5, fontface = 2, size = 9) +

geom_blank(data = AFRsample, mapping = aes(x = 8, y = 10)) +

coord_flip()

#Violin plots of IBI data (total sample) with overlayed boxplots and modal value lines

#y-axis has been restricted to 120 months for better data visualization

IBIplot <- ggplot() +

geom_violin(data = IBIsample %>% drop_na(Age.Group) %>% filter(Age.Group == "20-24" | Age.Group == "25-29") %>% filter(IBI > 8) %>% mutate(Income = factor(Income, levels = c("Natural Fertility", "Low", "Middle", "WIC Participant", "WIC Non-Participant"))), aes(x = Income, y = IBI, fill = Income), adjust = 3) +

geom_boxplot(data = IBIsample %>% drop_na(Age.Group) %>% filter(Age.Group == "20-24" | Age.Group == "25-29") %>% filter(IBI > 8) %>% mutate(Income = factor(Income, levels = c("Natural Fertility", "Low", "Middle", "High", "WIC Participant", "WIC Non-Participant"))), aes(x = Income, y = IBI), outlier.shape = NA, width = 0.1) +

xlab("") + ylab("IBI (months)") +

theme_classic(base_size = 20) +

theme(text = element_text(size = 25)) +

geom_bracket(xmin = "Low", xmax = "Middle", y.position = 120, label = "Country Income Groups", tip.length = c(0.31, 0.07), label.size = 6.2, coord.flip = TRUE) +

geom_bracket(xmin = "WIC Participant", xmax = "WIC Non-Participant", y.position = 120, label = "U.S. (2019)", tip.length = c(0.15, 0.17), label.size = 6.2, coord.flip = TRUE) +

scale_y_continuous(limits = c(0, 120), breaks = c(0, 20, 40, 60, 80, 100, 120)) +

geom_segment(mapping = aes(x = 0, xend = 7, y = 21, yend = 21), linetype = "longdash", color = "black", size = 0.9) +

geom_segment(mapping = aes(x = 0, xend = 7, y = 24, yend = 24), linetype = "longdash", color = "black", size = 0.9) +

geom_segment(mapping = aes(x = 0, xend = 7, y = 26, yend = 26), linetype = "longdash", color = "#009E73", size = 0.9) +

theme(legend.position = "none") +

scale_fill_manual(values = cbPalette2) +

annotate(geom = "text", label = "LI", x = 7.1, y = 28.1, angle = 40, size = 6) +

annotate(geom = "text", label = "NF & MI", x = 7.35, y = 27.6, angle = 40, size = 6) +

annotate(geom = "text", label = "U.S. WIC & U.S. NP", x = 7.58, y = 27.3, angle = 40, size = 6) +

annotate(geom = "text", label = "[b]", x = 8.3, y = 1, fontface = 2, size = 9) +

geom_blank(data = AFRsample, mapping = aes(x = 8.5, y = 10)) +

coord_flip()

#Arrange and save Figure 2

ggarrange(AFRplot, IBIplot, nrow = 2, ncol = 1)

ggsave("AFR.IBIplot.tiff", plot = last_plot(), device = tiff(), dpi = 400, width = 12, height = 22)
